# Genetic Risk Architecture of Late-Onset Alzheimer’s Disease: Mapping Single-Nucleotide Polymorphisms to Disease-Defining Molecular Pathways

**DOI:** 10.3390/life16101749

**Published:** 2026-10-18

**Authors:** Claudiu Matei, Nastaca Alina Palade, Ana-Maria Dobri-Nicoara, Dan Orga Dumitriu, Cosmin Mutu, Adriana Sorina Cristina Popescu, Alina-Liliana Pintea, Diter Atasie

**Affiliations:** 1 Department of Clinical Surgical, Faculty of Medicine, “Lucian Blaga” University of Sibiu, 550169 Sibiu, Romania; claudiu.matei@ulbsibiu.ro; 2 Medlife Polisano Hospital Sibiu, 550024 Sibiu, Romania; atasie.diter@ulbsibiu.ro; 3 Preclinical Department, Faculty of Medicine, “Lucian Blaga” University of Sibiu, 550169 Sibiu, Romania; 4 Department of Medical Genetics, Carol Davila University of Medicine and Pharmacy, 050047 Bucharest, Romania; anamaria.dobri@gmail.com; 5 Department of Clinical Medicine, Faculty of Medicine, “Lucian Blaga” University of Sibiu, 550169 Sibiu, Romania; dan.orgadumitriu@ulbsibiu.ro (D.O.D.); cosmin.mutu@ulbsibiu.ro (C.M.); adriana.popescu@ulbsibiu.ro (A.S.C.P.); 6 County Emergency Clinical Hospital Sibiu, 550025 Sibiu, Romania; 7 Department of Physical Medicine and Rehabilitation, Faculty of Medicine, “Lucian Blaga” University of Sibiu, 550169 Sibiu, Romania; alina-liliana.pintea@ulbsibiu.ro

**Keywords:** Alzheimer’s disease, late-onset Alzheimer’s disease, single-nucleotide polymorphisms (SNPs), genetic architecture, molecular pathways, neuroinflammation, amyloid-β, tau pathology

## Abstract

Late-onset Alzheimer’s disease (LOAD) is increasingly recognized as a complex, multifactorial disorder driven by the convergence of genetic susceptibility and interconnected molecular pathways, rather than a single linear pathogenic cascade. In this review, we integrate evidence from genome-wide association studies, sequencing analyses, and experimental research to map disease-associated single-nucleotide polymorphisms (SNPs) onto key biological processes underlying LOAD pathogenesis. Converging data consistently implicate a limited number of core domains, including amyloid-β metabolism, tau pathology, neuroinflammation, lipid homeostasis, endosomal–lysosomal trafficking, mitochondrial dysfunction, and synaptic impairment. Importantly, genetic risk variants are not randomly distributed, but cluster within these pathways, exerting coordinated effects across multiple biological systems and supporting a network-based organization of disease. We further examine how these pathways interact at a systems level, highlighting key interfaces such as the amyloid–tau, lipid–immune, inflammation–proteostasis, and mitochondria–synaptic dysfunction axes. These interconnected processes form self-reinforcing feedback loops that amplify neurodegeneration and contribute to disease progression. From a translational perspective, this integrated framework provides a mechanistic basis for the limited efficacy of single-target therapies and supports the development of multi-target and pathway-oriented strategies, as well as precision medicine approaches based on genetic and biomarker integration. Collectively, this review supports a unified model of LOAD as a polygenic network disorder and underscores the importance of pathway convergence in understanding disease heterogeneity and guiding future therapeutic development.

## 1. Introduction

Alzheimer’s disease (AD) represents one of the most pressing challenges in modern medicine, being the leading cause of dementia worldwide and a major global public health burden [1]. Late-onset Alzheimer’s disease (LOAD), defined by symptom onset after the age of 65 years, accounts for approximately 90% of all AD cases and arises from a complex interplay of aging, environmental exposures, and genetic susceptibility. By contrast, early-onset AD (EOAD) accounts for the remaining 5–10% of cases; although EOAD is often equated with familial disease, only a minority of EOAD cases are explained by autosomal-dominant mutations in *APP, PSEN1*, or *PSEN2*, and many are sporadic, with a substantial polygenic and *APOE*-related contribution [2,3]. In the context of increasing life expectancy, elucidating the biological mechanisms underlying LOAD has become essential for improving early risk prediction and enabling targeted therapeutic strategies.

Genetic susceptibility plays a central role in shaping individual risk for LOAD [4]. Heritability estimates suggest that a substantial proportion of disease risk is genetically determined, with the apolipoprotein E ε4 allele (*APOE* ε4) representing the strongest and most consistently replicated genetic risk factor identified to date [5]. However, *APOE* ε4 alone does not fully capture the genetic architecture of the disease. Over the past decade, large-scale genome-wide association studies (GWASs) and meta-analyses have substantially expanded the catalogue of AD-associated loci, identifying numerous common variants in genes such as *CLU*, *CR1*, *PICALM*, *BIN1*, *ABCA7*, *CD33*, *CD2AP*, *ADAM10*, *SPI1* and *SORL1*, as well as rare variants with larger effect sizes, including those in triggering receptor expressed on myeloid cells 2 (*TREM2*) [6,7,8,9]. Notably, these variants are not randomly distributed, but tend to cluster within biologically coherent systems implicated in AD pathogenesis.

Accordingly, the field is moving from simple locus discovery toward functional interpretation. The key question is no longer only which SNPs confer risk, but how these variants reshape disease-relevant pathways within specific cell types and brain regions. Single-cell and spatial transcriptomic approaches now provide a framework for interpreting AD risk genes in their cellular and anatomical contexts. These approaches have revealed plaque-associated glial niches, heterogeneous microglial and astrocytic states, and region-specific vulnerability. Integrating GWASs with these omics approaches may therefore help bridge the gap between inherited susceptibility and the cellular mechanisms that determine resilience or progression toward neurodegeneration [10].

Converging evidence indicates that AD-associated susceptibility loci map onto a limited number of interconnected molecular pathways, including lipid and cholesterol metabolism, amyloid-β (Aβ) production and clearance, tau phosphorylation and aggregation, microglial activation and neuroinflammatory processes, and endosomal–lysosomal trafficking. Additional processes, such as synaptic dysfunction and neurovascular impairment, including blood–brain barrier disruption, further contribute to disease progression [11,12,13]. Collectively, these findings support a model of pathway convergence, in which multiple genetic and molecular processes interact dynamically rather than operating as isolated pathogenic cascades [14]. Traditionally, AD has been conceptualized through linear pathogenic frameworks, most notably the amyloid cascade hypothesis [15]. While this model has provided important mechanistic insights, its explanatory scope remains incomplete, particularly in light of repeated therapeutic failures targeting solely the amyloid pathway [16]. Increasing genetic and experimental evidence instead supports a paradigm shift toward a systems-level perspective, in which LOAD is best understood as a network-based disorder [17]. Within this framework, diverse genetic variants, including single-nucleotide polymorphisms (SNPs), exert their effects through shared molecular circuits, leading to common downstream consequences such as impaired proteostasis, chronic neuroinflammation, and reduced synaptic plasticity [18,19].

**Figure 1:**
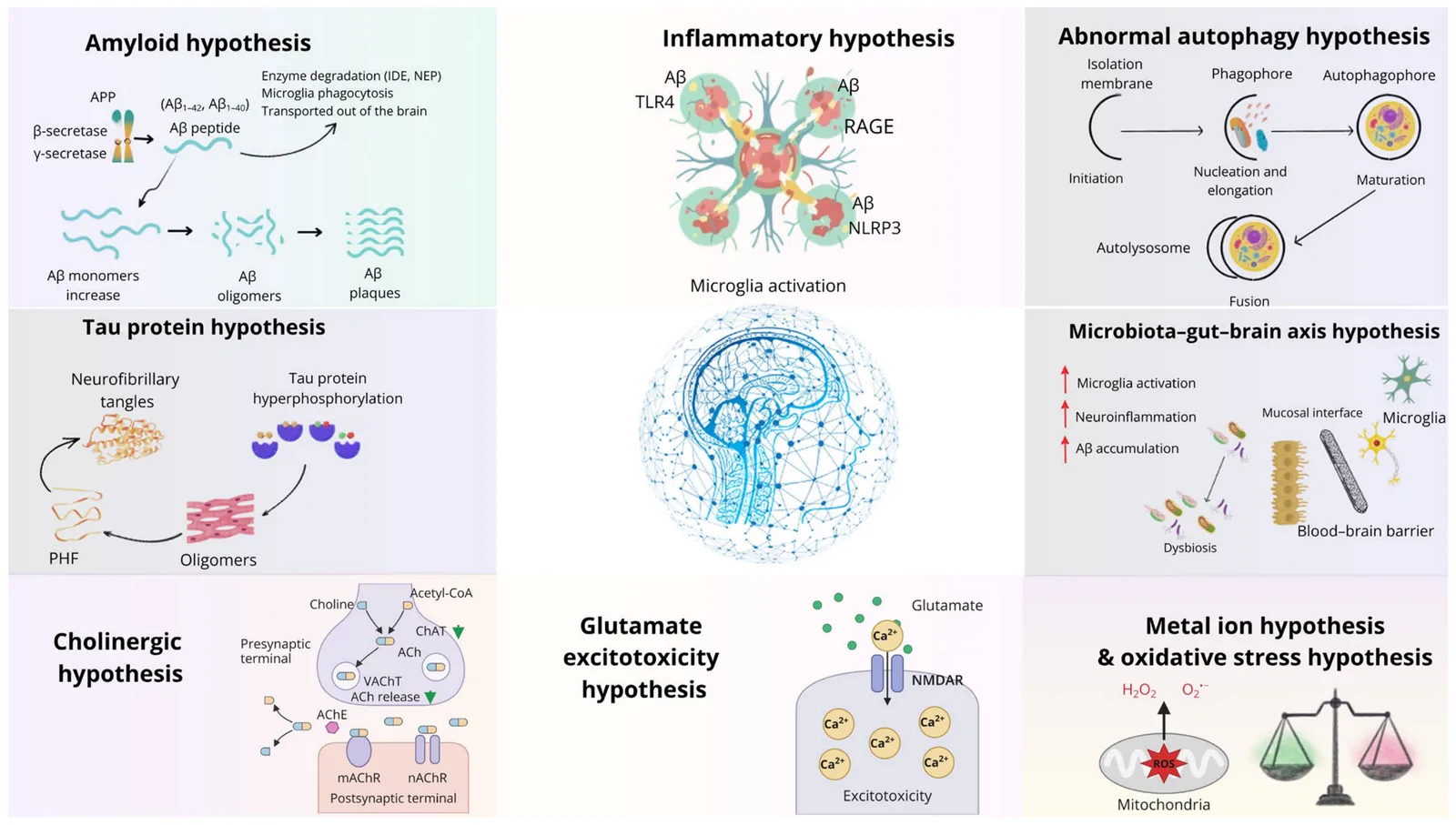
Major pathogenic hypotheses of Alzheimer’s disease. The panels summarize the amyloid, inflammatory, abnormal autophagy, tau, microbiota–gut–brain axis, cholinergic, glutamate excitotoxicity, and metal ion/oxidative stress hypotheses; the central network image symbolizes their interconnection. Black arrows indicate the sequence of pathological processes; red upward arrows indicate an increase and green downward arrows indicate a decrease. Colors are used for visual distinction only. ACh, acetylcholine; AChE, acetylcholinesterase; acetyl-CoA, acetyl coenzyme A; Aβ, amyloid-β; APP, amyloid precursor protein; ChAT, choline acetyltransferase; H_2_O_2_, hydrogen peroxide; IDE, insulin-degrading enzyme; mAChR, muscarinic acetylcholine receptor; nAChR, nicotinic acetylcholine receptor; NEP, neprilysin; NLRP3, NOD-like receptor protein 3; NMDAR, N-methyl-D-aspartate receptor; O_2_•^−^, superoxide anion; PHF, paired helical filaments; RAGE, receptor for advanced glycation end-products; ROS, reactive oxygen species; TLR4, Toll-like receptor 4; VAChT, vesicular acetylcholine transporter. Created with Canva (www.canva.com).

The multifactorial nature of AD is further reflected by the coexistence of multiple pathogenic hypotheses, as illustrated in Figure 1, which highlights their overlapping and interconnected mechanisms. These include the amyloid, tau, inflammatory, and abnormal autophagy hypotheses, which are examined in detail in this review, as well as the cholinergic, glutamate excitotoxicity, metal ion/oxidative stress, and microbiota–gut–brain axis hypotheses, which are shown for completeness but lie outside the genetic focus of the present work. Despite substantial advances, a major challenge remains the lack of integrative frameworks that systematically connect genetic susceptibility loci with coherent molecular pathways and clinically relevant disease mechanisms. Evidence linking individual AD-associated SNPs to cognitive decline, disease progression, and clinical outcomes remains heterogeneous and often fragmented [20,21]. Moreover, variability in population ancestry, study design, genotyping platforms, cognitive assessment tools, and statistical methodologies further complicates direct comparison and synthesis across studies [3,22,23].

Despite this progress, existing reviews have largely treated genetic risk loci and molecular pathways as parallel bodies of evidence rather than systematically mapping one onto the other at the level of individual variants. Building on this gap, the present review pursues three specific aims: (i) to catalogue LOAD-associated SNPs and assign each to its principal biological pathway based on current mechanistic evidence; (ii) to characterize the points of convergence and cross-talk between these pathways, with particular attention to the amyloid–tau, lipid–immune, inflammation–proteostasis, and mitochondria–synaptic axes; and (iii) to translate this pathway-level architecture into concrete implications for multi-target and precision-medicine therapeutic strategies. In doing so, this review moves beyond a purely descriptive catalogue of risk variants toward an explicit variant-to-pathway-to-therapy framework for LOAD. Accordingly, Section 3 outlines the genetic landscape of LOAD, Section 4 examines each core molecular pathway and its genetic determinants, Section 5 integrates these pathways into a systems-level framework, and Section 6 and Section 7 discuss the clinical, therapeutic, and research implications of this framework.

## 2. Literature Search Strategy and Scope

This narrative review was based on a structured literature search designed to identify and synthesize relevant evidence on the genetic architecture and molecular pathways involved in LOAD. Literature searches were performed in PubMed, Scopus, and Web of Science, with the final search update conducted in mid-2026.

The search strategy combined keywords and Medical Subject Headings (MeSH) terms related to “Alzheimer’s disease”, “late-onset Alzheimer’s disease”, “genetics”, “single-nucleotide polymorphisms”, “genome-wide association studies”, “neuroinflammation”, “amyloid-β”, “tau pathology”, and “lipid metabolism”, using Boolean operators (AND/OR) across fields. The search was limited to articles published in English between 2000 and 2026 to capture both foundational and recent advances in the field. Reference lists of key articles and prior reviews were additionally hand-searched to identify further relevant publications not captured by the electronic search. The final bibliography comprises 199 sources.

Both original research articles and review papers were considered eligible for inclusion. Selection prioritized studies with direct relevance to genetic susceptibility, molecular mechanisms, and pathway-level interactions in LOAD, with particular emphasis on genome-wide association studies (GWASs), meta-analyses, large cohort studies, and experimental research providing mechanistic insight. Priority was given throughout to peer-reviewed sources; a small number of preprints were included only where no peer-reviewed equivalent was yet available at the time of the search. Studies addressing exclusively early-onset Alzheimer’s disease (EOAD; onset before 65 years), whether autosomal-dominant familial forms caused by *APP*, *PSEN1*, or *PSEN2* mutations or sporadic early-onset cases, or reported in languages other than English were not prioritized for inclusion.

Large-scale GWAS meta-analyses have now identified more than 75 independent LOAD risk loci [24], and over 100 genes have been proposed as candidate risk genes; an exhaustive locus-by-locus treatment was therefore beyond the scope of this review. The genes discussed in detail and summarized in Table 1 were selected using the following criteria: (i) genome-wide significant association with LOAD (*p* < 5 × 10^−8^) replicated in at least one large GWAS meta-analysis [6,9,25,26], or, for rare variants, replicated association in sequencing studies [7,27]; (ii) availability of experimental evidence linking the gene or variant to a defined biological mechanism; and (iii) representation of each core pathway examined in this review, so that every pathway is illustrated by at least one well-characterized locus. A small number of candidate genes that have not reached genome-wide significance (*CALHM1*, *PIN1*, *IL6R*) were retained because they illustrate mechanisms that are otherwise poorly represented among GWAS loci; these are explicitly flagged in Table 1 and should be interpreted with caution. Loci with predominantly non-coding signals of an uncertain target gene were not discussed individually; a complete catalogue is available in [26].

Consistent with the narrative rather than systematic nature of this review, study selection followed a qualitative and iterative process, without a pre-registered protocol, formal dual-reviewer screening, or a standardized quality-appraisal instrument, and no quantitative flow of records identified/screened/included was tracked. This approach prioritizes conceptual synthesis and the integration of genetic findings into a systems-level framework of disease pathogenesis, an approach consistent with the aims and limitations inherent to narrative (non-systematic) reviews.

## 3. Genetic Landscape of Late-Onset Alzheimer’s Disease

The genetic landscape of LOAD is characterized by a complex and highly polygenic architecture, in which disease susceptibility arises from the cumulative effects of numerous genetic variants distributed across the genome rather than a single causative mutation. This architecture is predominantly driven by common variants with modest individual effect sizes, complemented by rarer variants with greater functional impact, which together contribute to disease risk, phenotypic heterogeneity, and variability in clinical presentation [3,28]. Such a distributed genetic framework highlights the limitations of single-variant approaches and underscores the need for integrative models that capture the combined influence of multiple loci [29].

At the core of this genetic landscape lies the *APOE* locus, which remains the most influential genetic determinant of LOAD. The ε4 allele confers a substantially increased risk of disease and is associated with an earlier age at onset in a dose-dependent manner. Beyond its epidemiological significance, *APOE* ε4 exhibits pleiotropic biological effects, influencing lipid transport, Aβ metabolism, synaptic function, and inflammatory signaling. This multifunctional role positions *APOE* as a central hub linking genetic susceptibility to multiple downstream pathogenic processes [21].

Advances in GWASs over the past decade have considerably expanded the number of loci associated with LOAD. Variants in genes such as *CLU*, *CR1*, *PICALM*, *BIN1*, *ABCA7, CD33, CD2AP,* and *SORL1*, together with rare coding variants in *TREM2*, have been consistently replicated across large-scale cohorts and meta-analyses [30,31,32,33,34]. Although these loci represent distinct genetic signals, they are not randomly distributed across the genome, but converge on a limited number of core biological pathways, including immune regulation, lipid homeostasis, and Aβ/tau processing [9,25]. A structured overview of representative genetic variants, their associated biological roles, and their mechanistic implications is presented in Table 1. The criteria used to select these representative loci are described in Section 2. Specifically, genes such as *TREM2*, *CD33,* and *CR1* are primarily linked to microglial activation and immune response, whereas *PICALM*, *BIN1*, and *SORL1* are implicated in vesicular trafficking and Aβ processing. Similarly, *APOE* and *ABCA7* play key roles in lipid metabolism and Aβ clearance, highlighting the intersection between metabolic and neurodegenerative pathways [33,35,36].

**Table 1:** Representative genetic variants associated with LOAD and their principal biological pathways.

Gene	SNP (Example)	Type	Pathway	Mechanism	Effect	Evidence	Refs.
*APOE*	rs429358/rs7412	Common	Lipid metabolism/Aβ clearance	Impaired cholesterol transport leading to reduced Aβ clearance and increased aggregation	Strong ↑ risk (ε4 allele)	GWAS, biomarker studies	[5,37]
*TREM2*	rs75932628	Rare	Neuroinflammation/microglial function	Impaired microglial activation and phagocytosis, reduced clearance of Aβ	Moderate–high ↑ risk	Sequencing studies	[7,36]
*CLU (ApoJ)*	rs11136000	Common	Lipid transport/proteostasis	Chaperone-mediated modulation of Aβ aggregation and clearance	Protective or risk-modifying	GWAS	[6,25]
*PICALM*	rs3851179	Common	Endocytosis/vesicular trafficking	Dysregulation of clathrin-mediated endocytosis and Aβ transport across BBB	Alters risk	GWAS	[25,33]
*BIN1*	rs744373	Common	Tau pathology/cytoskeleton	Modulates cytoskeletal dynamics and tau interaction, promoting tau pathology	↑ risk	GWAS + functional studies	[38]
*ABCA7*	rs3764650	Common/rare	Lipid metabolism/phagocytosis	Impaired lipid efflux and reduced microglial Aβ clearance	↑ risk	GWAS + sequencing	[37,39]
*CR1*	rs6656401	Common	Immune response/complement system	Dysregulation of complement cascade leading to enhanced inflammation	↑ risk	GWAS	[6]
*SORL1*	rs11218343	Rare/common	Endosomal trafficking/APP processing	Altered APP sorting leading to increased Aβ production	↑ risk	GWAS + sequencing	[32,39]
*CD33*	rs3865444	Common	Neuroinflammation	Inhibition of microglial Aβ uptake and clearance	↑ risk	GWAS	[31,40]
*CD2AP*	rs9349407	Common	Cytoskeleton/endocytosis	Disruption of synaptic and vesicular dynamics	↑ risk	GWAS	[31,40]
*EPHA1*	rs11767557	Common	Cell signaling/immune modulation	Modulation of immune signaling pathways affecting neuroinflammation	Modest ↑ risk	GWAS	[31,40]
*MS4A cluster*	rs983392	Common	Immune regulation	Regulation of microglial activation and immune response	↑ risk	GWAS	[31,40]
*ADAM10*	rs514049	Common	Non-amyloidogenic processing	α-secretase activity precluding Aβ formation	Protective/risk-modifying	GWAS + functional	[41,42]
*SPI1 (PU.1)*	rs1057233	Common	Myeloid/microglial regulation	Lowers PU.1 expression, delaying myeloid-driven disease onset	↓ risk	GWAS	[8]
*CALHM1*†	rs2986017	Common	Calcium signaling/Aβ clearance	Modulates neuronal Ca^2+^ homeostasis affecting Aβ levels	Alters risk	Functional + genetic	[43,44]
*MAPT*	rs242557	Common	Tau pathology	Influences tau expression and aggregation	Alters risk	GWAS	[34,45,46]
*PIN1*†	rs2233678	Common	Tau folding	Modulates tau conformational folding	Risk-modifying	Expression studies	[45]
*IL6R*†	rs2228145	Common	Neuroinflammation	Altered IL-6 pathway activity in AD brain	↑ risk	Candidate-gene + biomarker	[47]
*PLD3*	rs145999145	Rare	Lysosomal/mitochondrial function	mtDNA degradation, lysosomal dysfunction	↑ risk	Sequencing	[48,49]
*CASS4*	rs7274581	Common	Cytoskeletal/axonal transport	Disrupted intracellular/axonal transport	↑ risk	GWAS	[50,51]

Note: Loci were selected according to the criteria described in Section 2: (i) genome-wide significant and replicated association in large GWAS meta-analyses, or replicated rare-variant association in sequencing studies; (ii) availability of mechanistic evidence; and (iii) coverage of each core pathway discussed in this review. Entries marked † are candidate-gene associations that have not reached genome-wide significance and are included for mechanistic illustration only. The SNP shown is a representative (lead or commonly reported) variant rather than the only associated variant. Type refers to allele frequency (common, minor allele frequency ≥ 1%; rare, <1%). ↑, increased risk; ↓, decreased risk. Aβ, amyloid-β; ApoJ, apolipoprotein J; APP, amyloid precursor protein; BBB, blood–brain barrier; GWAS, genome-wide association study; IL-6, interleukin 6; mtDNA, mitochondrial DNA; PU.1, Spi-1 proto-oncogene transcription factor; SNP, single-nucleotide polymorphism.

Beyond locus-specific effects, the cumulative burden of multiple variants provides further support for a polygenic model of LOAD. Polygenic risk scores (PRSs) have emerged as a valuable tool for integrating the combined effects of multiple susceptibility variants, estimating genetic risk beyond *APOE* status by capturing the aggregate effect of non-*APOE* loci. Because *APOE* exerts a disproportionately strong influence on LOAD risk, it is typically modeled separately from, or alongside, genome-wide PRS rather than folded into it. The observation that a non-*APOE* genetic risk score retains predictive value even among *APOE* ε3/ε3 individuals further supports an independent contribution of non-APOE polygenic burden to AD-related pathology [52,53,54].

An alternative, oligogenic perspective has also been proposed as a counterpoint to the standard polygenic model. Zhang et al. showed that, for LOAD, risk prediction from common variants peaks when only a small number of highly significant loci are included, rather than improving continuously as more variants are added, with Bayesian estimates suggesting fewer than 100 common SNPs with non-zero effects on LOAD risk [55]. This finding does not contradict the polygenic model outright, but suggests that the effective genetic architecture may be more concentrated than genome-wide PRS approaches assume.

While pathway-based PRS is not yet clinically established, emerging evidence indicates that pathway-specific scores may provide a biologically interpretable framework for examining how distinct components of genetic risk relate to AD biomarkers, tau pathology, neuroimaging alterations, cognitive decline, and clinical status [56,57,58,59,60]. Their interpretation, however, remains constrained by variant-to-gene mapping strategies, pathway non-independence, ancestry-specific generalizability, and parameter-dependent methodological choices, and their incremental predictive value over global PRS appears limited [56].

## 4. Molecular Pathways in LOAD

**Figure 2:**
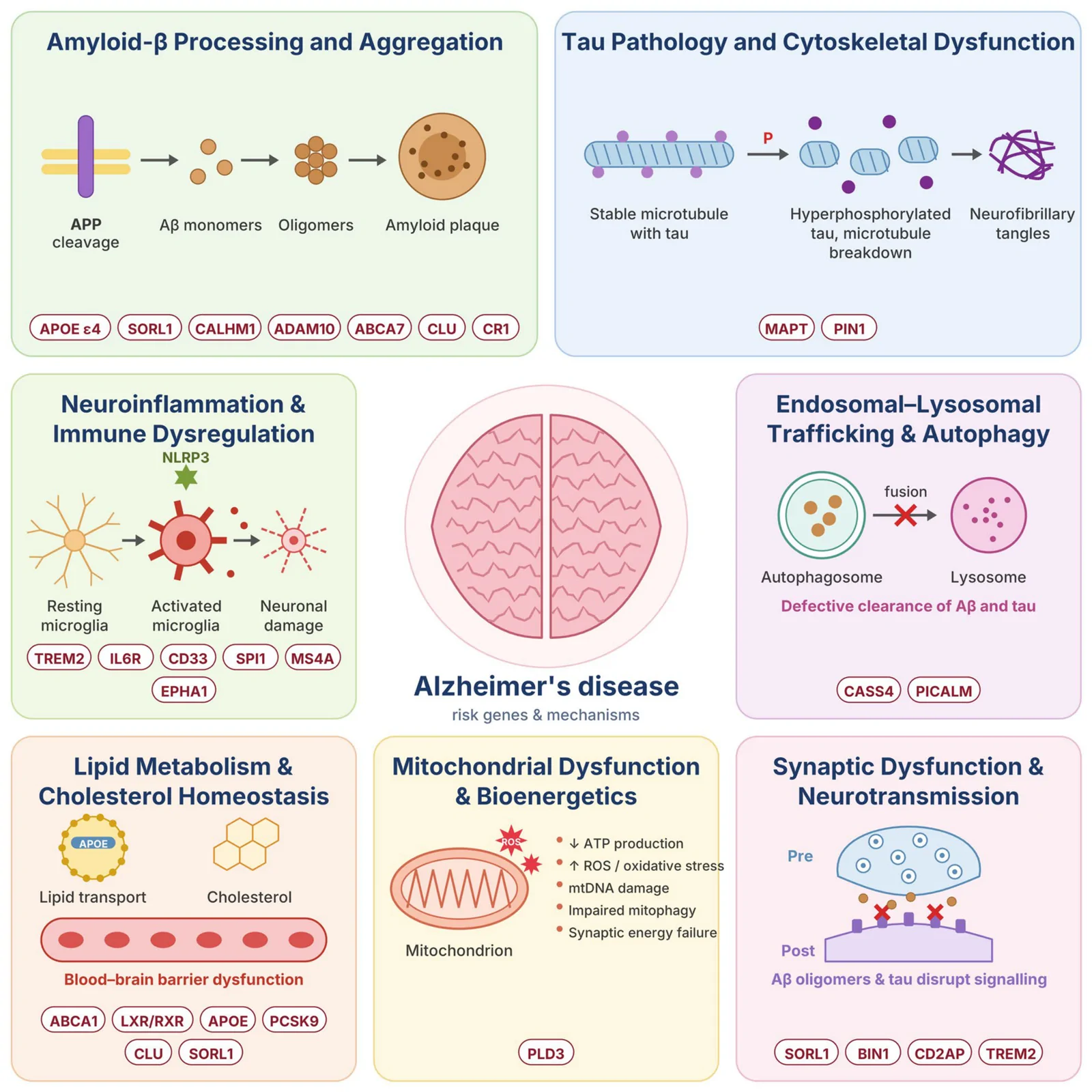
Core molecular pathways and associated risk genes in late-onset Alzheimer’s disease (LOAD). Each peripheral panel represents one pathway discussed in Section 4: amyloid-β processing, tau pathology, endosomal–lysosomal trafficking and autophagy, synaptic dysfunction, lipid metabolism, neuroinflammation, and mitochondrial dysfunction. Risk genes (red-outlined labels) are placed next to the pathway(s) they principally affect (see Table 1); genes shown more than once act on several pathways. Black arrows indicate the progression of pathological processes; red crosses indicate impaired or blocked processes; “P” and purple dots denote tau phosphorylation; ↑ and ↓ indicate an increase and a decrease, respectively. Panel colors are used for visual distinction only. Aβ, amyloid-β; APP, amyloid precursor protein; ATP, adenosine triphosphate; LXR/RXR, liver X receptor/retinoid X receptor; mtDNA, mitochondrial DNA; NLRP3, NOD-like receptor protein 3; Post, postsynaptic terminal; Pre, presynaptic terminal; ROS, reactive oxygen species. Created with Canva (www.canva.com).

LOAD risk loci converge on a limited number of core molecular pathways, each of which is examined in turn in the following subsections. These pathways, and the risk genes principally assigned to each of them, are summarized in Figure 2.

### 4.1. Amyloid-β Processing and Aggregation

Aβ biology occupies a central position in AD pathogenesis, encompassing the production, aggregation, clearance, and downstream neurotoxic effects of Aβ peptides. Despite ongoing debate, converging experimental, genetic, and biomarker evidence continues to support a key role for amyloid dysregulation as an early and influential event in disease development [61].

**Figure 3:**
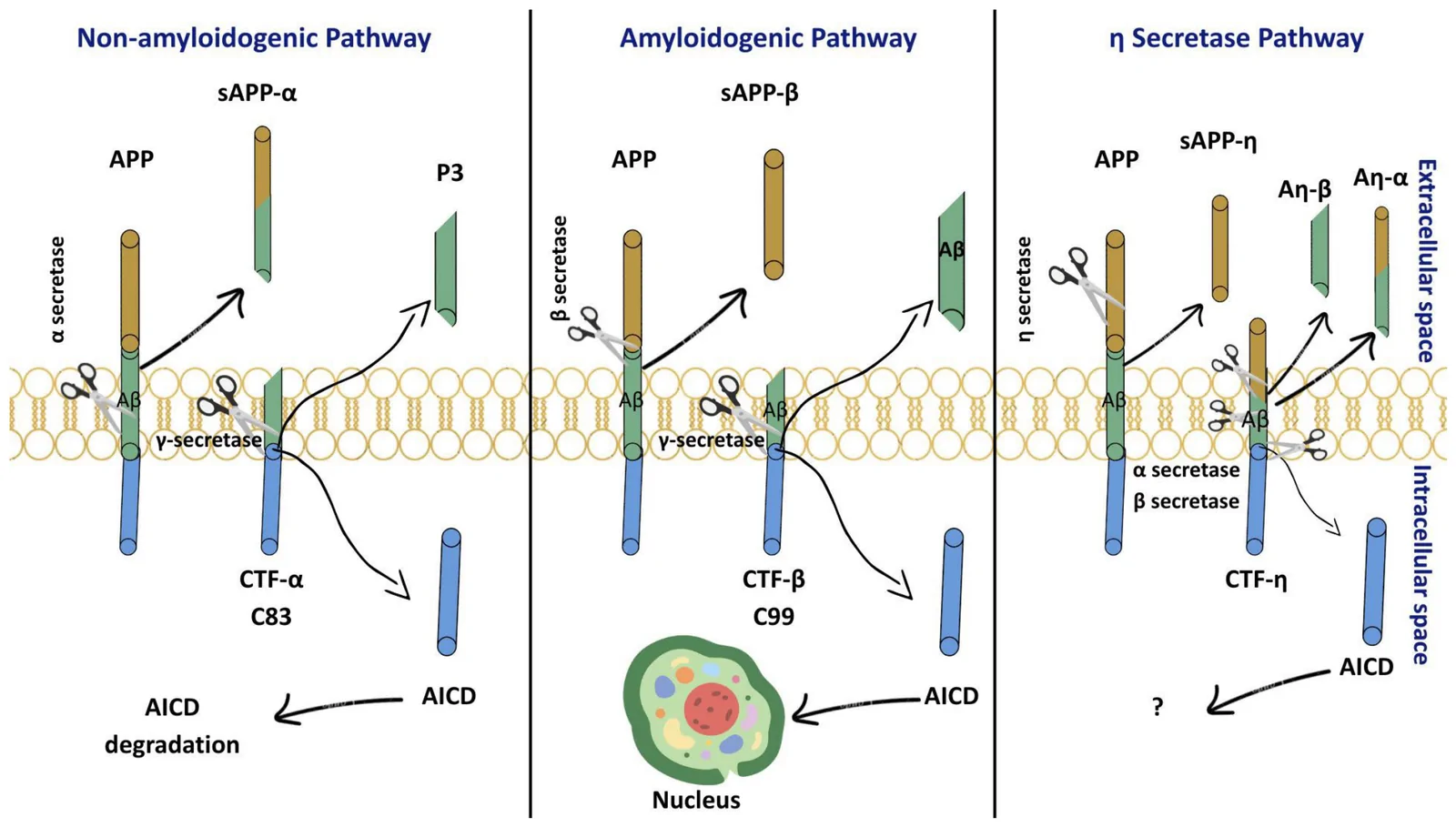
Proteolytic processing pathways of the amyloid precursor protein (APP). In the non-amyloidogenic pathway (left), α-secretase cleaves within the Aβ domain, precluding Aβ formation; in the amyloidogenic pathway (middle), sequential β- and γ-secretase cleavage releases Aβ; in the η-secretase pathway (right), APP cleavage generates Aη-α and Aη-β peptides. Scissors indicate secretase cleavage sites; straight arrows indicate the release of cleavage products, and curved arrows indicate the subsequent fate of the intracellular domain; “?” indicates that the fate of AICD in the η-secretase pathway is unknown. APP domains are color-coded: brown, extracellular N-terminal domain; green, Aβ region; blue, intracellular C-terminal domain. The lipid bilayer separates the extracellular (top) and intracellular (bottom) spaces. Aβ, amyloid-β; AICD, APP intracellular domain; Aη, amyloid-η; C83 and C99, 83- and 99-amino-acid C-terminal fragments; CTF, C-terminal fragment; P3, 3 kDa peptide; sAPP, soluble APP. Created with Canva (www.canva.com).

The amyloid cascade hypothesis remains one of the most extensively studied frameworks in AD, proposing that extracellular Aβ accumulation represents an initiating event that precedes tau pathology, synaptic dysfunction, and neurodegeneration. Aβ is generated through amyloidogenic processing of the amyloid precursor protein, involving sequential cleavage by β- and γ-secretases, which produces aggregation-prone peptides—particularly Aβ42—alongside the β-C-Terminal Fragment (βCTF) and APP Intracellular Domain (AICD). This pathway competes with the non-amyloidogenic α-secretase route, which precludes Aβ formation and is generally considered neuroprotective [41,42]. These APP processing routes are summarized in Figure 3. In addition to the canonical α- and β-secretase pathways, APP can be cleaved by η-secretase (membrane-type 5 matrix metalloproteinase), generating sAPP-η and the Aη-α and Aη-β peptides, which have been reported to impair hippocampal neuronal activity [62].

Beyond production, Aβ burden is determined by a dynamic equilibrium between generation and clearance. Impairments in clearance mechanisms—rather than overproduction alone—are increasingly recognized as a major driver of amyloid accumulation in sporadic LOAD. These processes are closely linked to lipid metabolism, vascular integrity, and immune-mediated clearance pathways [63,64,65]. Illustrating this link, a recent study demonstrated that BBB-targeted modulation of low-density lipoprotein receptor-related protein 1-mediated transcytosis enhances Aβ clearance from the brain in AD model mice. This intervention was further associated with reduced amyloid burden and improved cognitive performance, directly linking BBB transport mechanisms to amyloid pathology [66].

A key regulator of Aβ metabolism is APOE, whose isoforms exert differential effects on amyloid deposition and clearance. The *APOE* ε4 allele is consistently associated with increased cerebral Aβ burden, reduced clearance efficiency, and enhanced aggregation propensity compared with ε3 and ε2 isoforms. In addition to its direct effects on amyloid dynamics, APOE is functionally integrated within lipid metabolism pathways and contributes to vascular amyloid deposition. This pleiotropic role positions APOE as a central hub linking amyloid biology with lipid homeostasis and vascular dysfunction [67,68]. Importantly, amyloid pathology is tightly interconnected with neuroinflammatory processes: inflammatory signaling can enhance amyloidogenic processing, while Aβ accumulation activates microglia and astrocytes, amplifying chronic neuroinflammation and neuronal injury [69,70].

Genetic evidence further reinforces the central role of amyloid-related mechanisms in LOAD. Loss-of-function variants in *SORL1* are associated with decreased SORLA, which impairs lysosomal enzyme trafficking and degradation in human microglia-like cells, leading to defective clearance and lysosomal accumulation of amyloid-β and synaptic material [71,72,73]. Polymorphisms in *CALHM1* may influence Aβ homeostasis through modulation of neuronal calcium signaling [43,44]. Furthermore, GWAS-identified loci such as *ABCA7, CLU*, and *CR1* converge on lipid metabolism and immune-mediated clearance pathways [74,75]; in particular, *ABCA7* variants are associated with reduced microglial phagocytic capacity and impaired Aβ clearance and production, thereby promoting amyloid accumulation [76,77].

Rather than acting in isolation, the Aβ pathway functions as a central hub that integrates genetic susceptibility with multiple interconnected biological systems, including lipid metabolism, immune response, and vascular function—a convergence explored further in Section 5.

### 4.2. Tau Pathology and Cytoskeletal Dysfunction

Beyond amyloid dysregulation, tau pathology represents a second major and closely interconnected axis in AD, characterized by abnormal hyperphosphorylation, structural destabilization, and aggregation of tau protein into neurofibrillary tangles, ultimately leading to neuronal dysfunction and degeneration [78]. A recent commentary proposed that tau pathology may be a stronger predictor of neurodegeneration than amyloid burden, noting that baseline tau PET—unlike amyloid PET—closely mirrors the severity and regional pattern of subsequent brain atrophy; this interpretation positions tau as a more direct mediator of synaptic loss, neuronal injury, and clinical decline in AD, although it reflects a single expert perspective rather than a systematic comparative analysis [79].

Under physiological conditions, tau stabilizes microtubules and supports axonal transport. Disruption of the kinase–phosphatase balance leads to pathological hyperphosphorylation, reducing tau’s microtubule-binding capacity and promoting detachment, misfolding, and aggregation [80]. Consequently, tau dysregulation contributes directly to synaptic dysfunction [81]. Although tau hyperphosphorylation is often assumed to cause neuronal death through loss of microtubule binding and microtubule destabilization, this sequence remains insufficiently supported experimentally; current evidence more strongly favors a toxic gain-of-function model, in which disease-modified, soluble tau species directly promote neuronal degeneration independently of overt microtubule disassembly or filament formation [82].

Recent findings further refine this model. Neurofibrillary tangles are not homogeneous lesions but comprise distinct maturity-related immunophenotypes that progress with Braak stage; additionally, specific post-translational modifications, such as Arg-mediated tau Y394 phosphorylation, expand the range of molecular mechanisms potentially driving tau maturation and AD-related tauopathy [80,83,84]. Another key regulator of tau’s contribution to early neurodegeneration is GSK-3β activity, which modulates tau interaction with kinesin-1-dependent axonal transport, leading to vesicle and mitochondrial trafficking defects, synaptic dysfunction, and neuronal degeneration [46].

Tau pathology evolves through distinct stages that correlate with disease severity and progression patterns. While traditionally considered downstream of Aβ pathology, increasing evidence suggests a bidirectional interaction: Aβ accumulation promotes tau phosphorylation and propagation, while tau in turn mediates many downstream neurotoxic effects [78]. This interplay positions tau as a critical effector within the broader pathogenic network. Genetic factors further contribute to tau-related mechanisms: variations in the microtubule-associated protein tau (*MAPT*) gene and polymorphisms in *PIN1* may influence tau expression, folding, and aggregation [34,45,46].

Collectively, tau pathology functions as the principal executor of neurodegeneration in this network—translating upstream amyloid and inflammatory signals into synaptic loss, neuronal injury, and clinical decline.

### 4.3. Neuroinflammation and Immune Dysregulation

Neuroinflammatory signaling and microglial dysfunction are increasingly recognized as important and dynamic drivers of AD pathogenesis. Rather than representing a secondary response to neurodegeneration, accumulating evidence indicates that immune dysregulation is an integral component of disease initiation and progression [85]. Microglia, the resident immune cells of the central nervous system, exhibit remarkable heterogeneity and functional plasticity, adopting distinct activation states in response to disease stage and microenvironmental cues [86]. Recent advances in single-cell transcriptomics have identified disease-associated microglia and other specialized phenotypes characterized by stage- and region-specific gene expression profiles, enriched in pathways related to phagocytosis, lipid metabolism, and immune signaling [87,88]. This functional diversity reflects a dynamic spectrum ranging from protective to maladaptive microglial responses.

Among the key regulatory pathways, TREM2 signaling has emerged as a central modulator of microglial activity, influencing both phagocytic capacity and inflammatory responses and thereby regulating the clearance of Aβ and cellular debris. Experimental studies demonstrate that modulation of TREM2-dependent pathways can alter disease trajectory by enhancing debris clearance while limiting chronic inflammatory activation [89,90], establishing TREM2 as an interface between genetic susceptibility and immune-mediated disease mechanisms. Interest is also growing in soluble TREM2 as a CSF and plasma biomarker of microglial response dynamics across preclinical and diagnosed AD. A related but distinct receptor, the pro-inflammatory myeloid receptor TREM1, has also emerged as a subject of interest: in mouse models, TREM1 deficiency prevented age-related alterations in myeloid metabolism, inflammation, and hippocampal memory function, linking TREM signaling more broadly to aging-related immune dysfunction [91,92]. This evidence remains preclinical, and TREM1 is not yet established as a standalone staging biomarker in human disease.

In parallel, metabolic reprogramming has been identified as a key determinant of microglial function. Impairment of insulin signaling promotes a shift toward glycolytic metabolism, reducing the efficiency of cellular clearance mechanisms and contributing to sustained inflammatory activation [93]. Disruption of autophagic processes further exacerbates microglial dysfunction, promoting lipid accumulation and increasing the production of pro-inflammatory cytokines, thereby amplifying neurodegenerative processes and facilitating tau propagation [94,95].

Inflammasome activation represents another major inflammatory axis in AD. The NLRP3 inflammasome acts as a key mediator of microglia-driven inflammation, linking upstream signaling pathways such as TLR/NF-κB to the release of pro-inflammatory cytokines, including IL-1β [96]. Inhibition of NLRP3 has been shown to attenuate inflammatory responses and reduce pathological burden in experimental models, supporting its role as a potential therapeutic target [97], with additional inflammasome-related pathways further expanding the spectrum of innate immune mechanisms contributing to disease progression [98].

Genetic evidence strongly reinforces the central role of immune pathways in LOAD susceptibility. GWASs have identified numerous risk loci that map to immune-related processes, indicating that neuroinflammation is genetically encoded within the disease architecture [9,99]. Variants in genes such as *TREM2*, as well as immune-related loci including *IL6R*, have been associated with altered inflammatory signaling that might influence disease risk [47,100].

Neuroinflammation is tightly interconnected with other pathogenic processes in AD: Aβ accumulation activates microglia and triggers inflammatory signaling, while chronic inflammation in turn promotes amyloidogenic processing and facilitates tau phosphorylation and propagation [101]. This bidirectional interaction integrates neuroinflammation within the broader network of AD pathology, consistent with a self-amplifying pathogenic loop in which microglia act as both key effectors and promising therapeutic targets [102].

### 4.4. Endosomal–Lysosomal Trafficking, Proteostasis, and Autophagy

Cellular homeostasis in LOAD critically depends on the integrity of endosomal–lysosomal trafficking and autophagic pathways, which collectively regulate protein sorting, recycling, and degradation. Disruption of these interconnected systems leads to a failure of proteostasis, resulting in the intracellular accumulation of misfolded and aggregation-prone proteins and contributing directly to neurodegeneration [103].

Endosomal compartments play a central role in intracellular trafficking and represent key sites for amyloid precursor protein processing. Internalized amyloid precursor protein is preferentially cleaved within endosomes, where the acidic environment favors β-secretase activity and promotes amyloidogenic processing. Consequently, alterations in endosomal trafficking can shift processing toward increased Aβ production, establishing a direct mechanistic link between trafficking defects and amyloid pathology. In parallel, lysosomal dysfunction impairs degradative capacity, limiting the clearance of aggregated proteins and leading to progressive cellular stress and toxicity [104,105].

Genetic evidence strongly supports the involvement of these pathways in LOAD susceptibility. Variants in *SORL1* are associated with impaired trafficking and increased amyloidogenic processing [9,106]. Similarly, loci such as *TREM2* and *CR1* intersect with endocytic and immune-related mechanisms, highlighting the integration of vesicular trafficking with microglial function and inflammatory signaling [103,107,108]. In addition, PLD3 has been implicated in AD pathogenesis through regulation of mitochondrial DNA degradation, lysosomal function, and APP metabolism, with its dysfunction linked to reduced degradation efficiency and accumulation of pathological substrates [48,49].

Autophagy represents a complementary and essential clearance mechanism responsible for the degradation of long-lived proteins and damaged organelles. Impairment of autophagic flux leads to defective clearance of protein aggregates, exacerbating intracellular toxicity and contributing to both Aβ and tau accumulation [109]. AD-associated loci such as *CASS4* further implicate cytoskeletal and trafficking-related mechanisms in disease pathogenesis, potentially reflecting disrupted intracellular or axonal transport pathways [6,50,51].

Dysfunction of these systems does not occur in isolation. Impaired trafficking enhances Aβ production, defective clearance mechanisms promote the accumulation of both Aβ and hyperphosphorylated tau, and lysosomal and autophagic dysfunction can amplify neuroinflammatory responses. Endosomal–lysosomal trafficking and autophagy therefore function as central regulators of proteostasis in LOAD, linking genetic susceptibility to the accumulation of neurotoxic protein species and to broader network-level dysfunction [109,110,111].

### 4.5. Lipid Metabolism and Cholesterol Homeostasis

Lipid and cholesterol metabolism represent a critical regulatory layer in LOAD, influencing membrane composition, cellular signaling, and neuronal resilience. Within the central nervous system, cholesterol plays an essential role in maintaining synaptic integrity, membrane fluidity, and receptor organization; disruption of lipid homeostasis has profound consequences for neuronal stability, particularly through its effects on membrane architecture and signaling microdomains [112,113,114,115]. Alterations in lipid composition affect lipid-raft-dependent platforms that regulate protein trafficking and receptor-mediated signaling. These microdomains play a key role in amyloid precursor protein processing, as both β- and γ-secretase activities are enriched within cholesterol-rich membrane regions. Consequently, disturbances in cholesterol homeostasis can shift processing toward the amyloidogenic pathway, linking lipid metabolism directly to Aβ production and aggregation [114,116]. A recent line of research suggests that Alzheimer’s disease risk genes may converge on a common microglial lipid-droplet phenotype: *APOE4* promotes lipid-droplet-accumulating, neurotoxic microglia capable of inducing tau phosphorylation, while *PICALM* risk variation impairs microglial uptake of Aβ and myelin debris and shifts cells toward cholesterol synthesis and lipid-droplet formation [117,118].

APOE functions as the major apolipoprotein responsible for lipid transport in the brain, mediating cholesterol redistribution between astrocytes and neurons (Table 2). *APOE* ε4 is associated with reduced lipid transport efficiency, intracellular lipid accumulation, and altered membrane composition, all of which contribute to increased neuronal vulnerability [119,120,121]. In addition to its effects on lipid transport, *APOE* ε4 also impairs Aβ clearance and promotes aggregation. Recent studies using induced pluripotent stem cell-derived models have demonstrated that *APOE* ε4 induces lipid dysregulation, including impaired cholesterol efflux, accumulation of lipid droplets, and disrupted astrocyte–neuron metabolic coupling [122]. These findings suggest that lipid imbalance is not merely a secondary consequence but an intrinsic driver of cellular dysfunction in LOAD.

Genetic evidence further supports the central role of lipid metabolism in LOAD susceptibility, with variants in *ABCA1*, *PCSK9*, *CLU*, and *ABCA7* reinforcing the link between lipid transport, immune function, and cellular maintenance pathways [123,124,125,126].

**Table 2:** Functional roles of lipid metabolism-related genes in LOAD.

Gene	Molecular Function	Cellular Mechanism	Impact on AD-Related Processes	Relevance in LOAD	Refs.
*APOE* (ε4)	Lipid transport	Impaired lipid redistribution	↓ Aβ clearance, ↑ aggregation, altered membrane composition	Strongest genetic risk factor	[119,120,121]
*ABCA1*	Cholesterol efflux	Reduced APOE lipidation	Impaired Aβ clearance, disrupted membrane homeostasis	Aβ-clearance modifier	[126]
*ABCA7*	Lipid transport & phagocytosis	Impaired lipid handling and microglial function	Reduced Aβ clearance, increased inflammation	Increased susceptibility	[76,127]
*CLU (ApoJ)*	Lipid binding & chaperone	Extracellular chaperone-mediated suppression of protein aggregation	Modulates Aβ aggregation and clearance	Context-dependent role	[124,128]
*PCSK9*	LDL receptor regulation	Alters systemic lipid turnover	Indirect effects on brain lipid homeostasis	Potential modifier	[125]
LXR/RXR	Transcriptional regulation	Controls lipid metabolism genes (e.g., ABCA1)	Regulates cholesterol homeostasis and Aβ clearance	Regulatory dysfunction	[129]
*SORL1*	Endosomal trafficking	APP trafficking at the intersection with lipid-sorting pathways	Alters APP processing and Aβ production	Contributes to amyloid pathology	[9,106]

Note: ↑, increased; ↓, decreased. Aβ, amyloid-β; ApoJ, apolipoprotein J; APP, amyloid precursor protein; LDL, low-density lipoprotein; LXR/RXR, liver X receptor/retinoid X receptor.

### 4.6. Synaptic Dysfunction and Neurotransmission

Synaptic dysfunction represents one of the earliest and most clinically relevant features of LOAD, closely associated with cognitive decline and memory impairment [130]. Synaptic failure precedes overt neurodegeneration and serves as a key determinant of disease progression, with the degree of synaptic loss correlating more closely with cognitive impairment than either plaque or tangle burden alone.

Aβ oligomers play a central role in early synaptic impairment by disrupting receptor trafficking, postsynaptic signaling, and long-term potentiation [131]. These effects occur prior to plaque formation, highlighting the importance of soluble Aβ species—rather than insoluble deposits—in early synaptic toxicity [130,132]. Tau pathology further exacerbates synaptic dysfunction through its interaction with synaptic vesicle machinery: tau binds synaptic vesicles via Synaptogyrin-3, a vesicle-associated membrane protein involved in neurotransmitter release, thereby disrupting vesicle mobility and impairing presynaptic neurotransmitter release [133,134].

Neuroinflammatory processes also contribute to synaptic loss. Dysregulated microglial activity has been linked to excessive synaptic pruning, most notably through complement-mediated tagging of synapses for microglial engulfment, providing a mechanistic bridge between immune activation and synaptic degeneration [130]. Genetic evidence further supports the involvement of synaptic pathways in LOAD, with risk loci such as *CD2AP*, *BIN1*, and *SORL1* influencing synaptic structure and function [135,136,137].

Synaptic dysfunction thus constitutes a convergence point at which amyloid pathology, tau aggregation, immune dysregulation, and intracellular trafficking defects intersect, offering one of the most direct mechanistic links between molecular pathology and cognitive decline in LOAD.

## 5. Pathway Interactions and Systems-Level Integration in LOAD

### 5.1. Amyloid–Tau Axis

Few interactions in AD pathogenesis are as consequential as the interplay between amyloid and tau. A substantial body of evidence indicates that soluble amyloid-β oligomers, rather than insoluble plaque deposits alone, represent the primary neurotoxic species. These oligomers disrupt synaptic function, alter membrane integrity, induce calcium dyshomeostasis, and promote mitochondrial dysfunction and oxidative stress, thereby establishing a permissive environment for subsequent tau pathology [138,139,140]. Tau is a microtubule-associated protein that, under physiological conditions, stabilizes axonal microtubules. In Alzheimer’s disease, tau undergoes pathological hyperphosphorylation and conformational changes that lead to its detachment from microtubules, mislocalization to the somatodendritic compartment, and progressive aggregation into soluble oligomers, paired helical filaments, and NFTs, compromising cytoskeletal integrity and axonal transport and contributing directly to neuronal dysfunction and cell death [141,142].

The relationship between Aβ and tau is generally described as hierarchical, though the two processes are functionally interdependent rather than strictly sequential. Aβ accumulation is commonly considered an upstream event that facilitates tau pathology, in part through activation of the kinase GSK-3β, which enhances tau phosphorylation and aggregation; conversely, AMPK activity has been shown to counteract this process in some experimental contexts, with reduced AMPK signaling promoting tau hyperphosphorylation and AMPK upregulation ameliorating it [143].

This relationship is not uniformly protective, however: AMPK activation under conditions of acute metabolic stress has also been reported to directly phosphorylate tau at specific sites, suggesting that its net effect on tau pathology may depend on cellular energy state and disease stage rather than acting as a simple unidirectional safeguard [144]. In addition to its intracellular toxicity, pathological tau exhibits prion-like properties, enabling trans-synaptic propagation across interconnected neuronal networks. Misfolded tau species can be released into the extracellular space and internalized by neighboring neurons, where they seed further aggregation. This mechanism underlies the stereotypical spatiotemporal progression of neurodegeneration observed in Alzheimer’s disease and highlights the role of tau in network-level dysfunction [145,146]. The amyloid–tau axis is further amplified by neuroinflammatory mechanisms. Aβ accumulation activates microglia, initially enhancing phagocytic clearance but ultimately leading to chronic activation and functional impairment. Sustained microglial activation, together with NLRP3 inflammasome signaling and pro-inflammatory cytokine release, promotes both Aβ deposition and tau hyperphosphorylation. Moreover, microglia-derived extracellular vesicles have been implicated in the dissemination of tau pathology, reinforcing the interplay between amyloid, tau, and innate immune responses [143,147]. Moreover, recent data show that amyloid-β may efficiently drive tau pathology only in a permissive neuroimmune environment, where microglial activation modulates Aβ-dependent astrocyte reactivity, and reactive astrocytes in turn mediate the transition from Aβ accumulation to phosphorylated tau increase and AD-like tau deposition [148,149].

Metabolic dysregulation also contributes to the integration of amyloid and tau pathology within a broader systems-level framework. Insulin resistance and altered energy metabolism activate signaling pathways, including GSK-3β and c-Jun N-terminal kinase (JNK), that simultaneously enhance amyloidogenic processing of APP and tau phosphorylation. These findings support the concept that systemic metabolic disturbances can potentiate the amyloid–tau axis and accelerate disease progression [150,151,152,153]. Recent advances in biomarker research further substantiate the biological coupling between Aβ and tau. Plasma phosphorylated tau species, particularly p-tau217, have demonstrated strong associations with amyloid deposition in early disease stages and subsequently reflect tau accumulation and progression [154,155]. A novel plasma biomarker, eMTBR-tau243, has shown stronger association with tau-PET in intermediate and late Braak-region tau accumulation, though this finding awaits independent validation [144]. In sum, the amyloid–tau axis functions predominantly as a self-reinforcing pathogenic loop in which Aβ initiates a cascade of synaptic, metabolic, and inflammatory dysfunction while tau mediates much of the downstream neurodegeneration—while recognizing that Aβ-independent tau pathways, as in PART, indicate this axis is not the sole route to tau pathology in the aging brain.

### 5.2. Lipid–Immune Axis

Disturbances in lipid metabolism rarely act in isolation from the innate immune response in LOAD, and their interface constitutes one of the best-characterized genetic convergence points in the disease. Genetic studies have consistently identified key susceptibility genes involved in both lipid handling and microglial function, including *APOE*, *TREM2*, and *ABCA7*, highlighting this mechanistic overlap [40,156,157]. *APOE*, particularly the ε4 isoform, is the strongest genetic risk factor for LOAD and plays a central role in cholesterol transport, lipid homeostasis, and amyloid-β metabolism. *APOE* isoforms differentially regulate Aβ aggregation and clearance, with *APOE* ε4 promoting increased deposition and reduced clearance efficiency. APOE also directly modulates microglial activation states, shifting microglia toward a pro-inflammatory and less phagocytic phenotype, thereby linking lipid dysregulation to impaired immune responses [158].

TREM2 further exemplifies the intersection between lipid sensing and immune activation. TREM2 functions as a lipid sensor in microglia, regulating their metabolic fitness, survival, and phagocytic activity. Loss-of-function variants in *TREM2* impair microglial responses to lipid-rich debris and amyloid plaques, resulting in reduced plaque-associated microglial clustering and diminished Aβ clearance; TREM2 signaling has also been shown to interact functionally with APOE pathways [36,159]. ABCA7 contributes to this axis through its role in cholesterol efflux and phagocytosis: genetic variants in *ABCA7* are associated with impaired lipid transport and reduced clearance of Aβ, leading to enhanced plaque accumulation and inflammatory activation [127,160]. At the cellular level, microglia under pathological conditions accumulate intracellular lipid droplets, associated with reduced phagocytic capacity and increased production of pro-inflammatory cytokines, sustaining neuroinflammation and impairing clearance of toxic protein aggregates [94,161].

The role of cholesterol metabolism in amyloidogenic processing remains an active and genuinely unresolved area of investigation, and the relationship between cholesterol and Alzheimer’s disease is disputed in the literature. Current evidence shifts attention from plasma cholesterol as a risk factor toward brain cholesterol metabolism, where Aβ overload may inhibit cholesterol biosynthesis, reduce neuronal and glial cholesterol content, and thereby promote amyloid and tau pathology [162]. This axis operates through a bidirectional feedback loop: lipid accumulation promotes microglial activation and inflammatory signaling, while chronic inflammation further disrupts lipid metabolism, reinforcing a self-sustaining cycle. Systemic dyslipidemia and metabolic dysfunction further modulate this axis by influencing central nervous system lipid homeostasis and immune responses, amplifying neuroinflammation and disease progression [163].

Taken as a whole, this axis illustrates how genetic susceptibility, lipid metabolism, and innate immune dysfunction converge to drive impaired Aβ clearance and chronic neuroinflammation in LOAD.

### 5.3. Inflammation–Proteostasis Axis

Chronic neuroinflammatory activity and protein-clearance failure are mutually reinforcing processes in LOAD rather than sequential events. Increasing evidence suggests that sustained activation of innate immune pathways not only reflects disease progression but also actively disrupts proteostatic mechanisms, including autophagy, lysosomal degradation, and protein clearance systems [164]. Proteostasis—the dynamic regulation of protein synthesis, folding, trafficking, and degradation—is essential for neuronal integrity; in AD, this balance is disrupted, leading to the accumulation of misfolded proteins such as amyloid-β and hyperphosphorylated tau. The endosomal–lysosomal system and autophagic pathways normally clear these toxic species, but their dysfunction results in impaired degradation and intracellular accumulation of protein aggregates [165]. Neuroinflammation significantly exacerbates this failure: microglial activation, initially protective, becomes maladaptive during chronic stimulation, leading to sustained release of pro-inflammatory cytokines such as IL-1β, IL-6, and TNF-α, which interfere with autophagic flux and lysosomal function [95,166].

A key molecular link between inflammation and proteostasis is the NLRP3 inflammasome. Its activation in microglia leads to the maturation and release of IL-1β, which promotes amyloid deposition and tau pathology, while also impairing autophagic processes and contributing to lysosomal dysfunction [167,168].

The ubiquitin–proteasome system (UPS) provides a second, complementary layer of proteostatic control that is compromised in Alzheimer’s disease: its dysfunction leads to the accumulation of ubiquitinated proteins and contributes to the formation of neurofibrillary tangles and amyloid pathology, and inflammatory mediators can further impair proteasomal activity, linking immune activation with defective protein degradation [169].

This axis is therefore best understood as bidirectional and self-amplifying: chronic neuroinflammation impairs protein clearance mechanisms, while proteostatic failure sustains inflammatory activation, together driving the progressive accumulation of toxic protein species in LOAD.

### 5.4. Mitochondria–Synaptic Dysfunction Axis

Bioenergetic failure and synaptic impairment are tightly coupled in LOAD, and this coupling appears early in the disease course. Neurons are highly energy-dependent cells, and mitochondrial integrity is essential for maintaining synaptic transmission, plasticity, and overall neuronal survival; early mitochondrial dysfunction precedes overt neurodegeneration and contributes directly to synaptic failure [170,171]. Mitochondria play a central role in ATP production, calcium buffering, and regulation of reactive oxygen species (ROS). In LOAD, multiple studies have demonstrated impaired mitochondrial respiration, reduced ATP synthesis, increased oxidative stress, and disrupted mitochondrial dynamics, compromising neuronal energy supply and increasing vulnerability to excitotoxic and metabolic stress [172].

Amyloid-β and tau pathology further exacerbate mitochondrial dysfunction. Aβ accumulates within mitochondria, where it interacts with mitochondrial proteins such as amyloid-binding alcohol dehydrogenase (ABAD), impairing enzymatic function and increasing ROS production; pathological tau, in turn, disrupts mitochondrial transport along axons and dendrites, impairing the delivery of mitochondria to synaptic terminals where energy demand is highest [173,174,175]. As discussed in Section 4.6, synaptic dysfunction is among the earliest clinically relevant features of LOAD; here, mitochondrial impairment leads to insufficient ATP supply at synapses, disrupting neurotransmitter release, synaptic vesicle recycling, and postsynaptic signaling, while mitochondrial calcium dysregulation further contributes to excitotoxicity and synaptic loss [171,172].

Oxidative stress further amplifies this axis: excessive ROS production damages lipids, proteins, and DNA, including mitochondrial DNA (mtDNA), leading to a decline in mitochondrial function and further ROS generation—a vicious cycle that accelerates synaptic degeneration and neuronal loss [170]. Mitochondrial dysfunction is also closely linked to impaired proteostasis and neuroinflammation: defective mitophagy leads to the accumulation of damaged mitochondria, which release danger-associated molecular patterns (DAMPs) that activate microglia and promote inflammatory responses, which in turn further impair mitochondrial function [176,177].

This axis thus operates as a self-reinforcing driver of neuronal failure in LOAD, positioning mitochondrial and synaptic resilience as a plausible therapeutic target, discussed further in Section 6.

### 5.5. Convergence of Genetic Risk into Shared Pathways

The four axes described above do not operate as independent silos—they are the product of a single, highly polygenic genetic architecture. LOAD susceptibility loci exert modest individual effects but converge onto a limited number of interconnected biological pathways: amyloid processing, lipid metabolism, innate immunity, proteostasis, and mitochondrial function, ultimately shaping a unified pathogenic network [9,26,178]. GWASs have consistently identified risk variants in genes such as *APOE*, *TREM2*, *ABCA7*, *CLU*, *PICALM*, and *BIN1*, which cluster within functional pathways rather than discrete molecular events. Many of these genes are highly expressed in microglia and involved in lipid sensing, phagocytosis, and immune regulation, reinforcing the centrality of the lipid–immune axis in disease susceptibility [179,180].

Critically, these genetic factors do not map exclusively to single pathways but exert pleiotropic effects across multiple biological systems: *APOE* regulates lipid transport, amyloid aggregation, and microglial activation; *TREM2* integrates lipid sensing with immune signaling and phagocytic function; and *ABCA7* influences both cholesterol efflux and amyloid clearance [181,182]. This convergence is particularly evident at the interface between amyloid pathology and immune activation, where several risk loci modulate microglial responses to amyloid deposition, influencing plaque clearance, inflammatory signaling, and disease progression [183]. Genetic factors similarly intersect with proteostasis pathways, including endosomal–lysosomal trafficking and autophagy: genes such as *PICALM* and *BIN1* are involved in vesicular trafficking and membrane dynamics, influencing both APP processing and tau propagation, and their disruption contributes to impaired protein clearance and proteostatic failure [184].

Mitochondrial function and synaptic integrity are shaped more indirectly by genetic risk. Although fewer GWAS loci map directly to mitochondrial genes, many risk variants influence upstream processes—lipid metabolism, inflammation, proteostasis—that in turn affect mitochondrial homeostasis; the resulting oxidative–inflammatory–bioenergetic loop is well characterized even where its upstream genetic drivers remain less directly mapped [171,172,185]. Mitochondrial dysfunction in Alzheimer’s disease therefore often arises as a downstream consequence of genetically driven perturbations elsewhere in the network.

At a systems level, this produces a highly interconnected network with feedback loops and cross-talk between processes: amyloid accumulation triggers immune activation; inflammation impairs proteostasis; defective protein clearance enhances tau pathology; and mitochondrial dysfunction amplifies both oxidative stress and synaptic failure—with genetic risk factors contributing to each node and thereby shaping both disease initiation and progression [26]. This framework supports a shift from a linear “amyloid cascade” model toward a multidimensional network model of Alzheimer’s disease, providing a more comprehensive account of disease heterogeneity and underscoring the value of targeting multiple pathways simultaneously—a rationale developed further in Section 6 [3,184].

## 6. Clinical and Therapeutic Implications

### 6.1. Why Single-Target Therapies Fail

**Figure 4:**
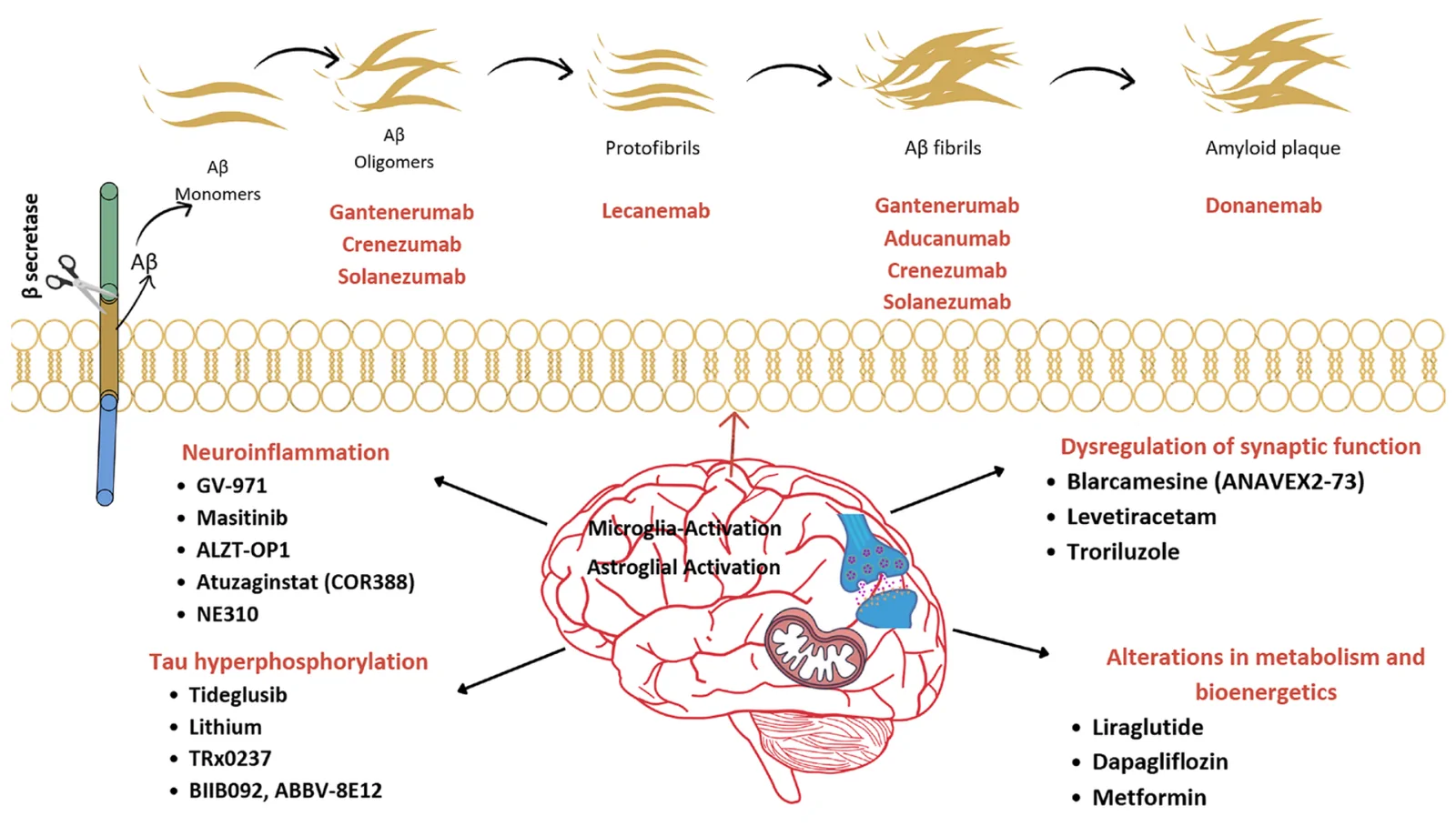
Therapeutic targets and representative disease-modifying strategies in Alzheimer’s disease. Top: anti-amyloid antibodies (red) positioned by the Aβ species they target, from monomers to plaques (gold); black arrows indicate the progressive aggregation of Aβ following β-secretase cleavage of APP at the cell membrane. Bottom: investigational agents (black) grouped by therapeutic target category (red headings), namely neuroinflammation, tau hyperphosphorylation, synaptic dysfunction, and metabolism/bioenergetics; arrows point from the brain to each target category. Agents differ in clinical-development status. Aβ, amyloid-β; APP, amyloid precursor protein. (www.canva.com).

One of the central lessons from AD drug development is that targeting a single pathogenic process rarely produces substantial clinical benefit. This is particularly evident in anti-amyloid programs (Figure 4). Although agents such as lecanemab reduce amyloid burden and produce measurable but modest slowing of cognitive decline [186], several earlier and contemporaneous anti-amyloid strategies have failed to deliver meaningful clinical benefit despite biomarker effects. Trials of bapineuzumab, solanezumab, semagacestat, and gantenerumab illustrate that reduction in a single pathological feature does not necessarily translate into robust disease modification [187].

Several factors likely explain these limitations. First, AD pathology is already biologically advanced by the time of clinical diagnosis, with amyloid, tau, inflammatory activation, synaptic injury, and vascular dysfunction often already established [188]. Second, individual therapeutic targets may lie upstream of some disease processes but downstream of others, limiting the impact of isolated interventions, a challenge also described for multi-target drug design in other complex diseases such as cancer [189]. Third, genetic and clinical heterogeneity means that not all patients are driven by the same dominant mechanisms, paralleling heterogeneity-driven treatment resistance described in oncology [190]. Finally, interventions directed at a single pathway may leave intact the feedback loops that sustain disease progression, such as the reciprocal interactions between Aβ and tau, inflammation and proteostasis, or lipid dysregulation and microglial dysfunction [26].

Taken together, these findings suggest that the failure of many single-target approaches is not merely a pharmacological issue, but a conceptual one. AD is unlikely to be durably modified by interventions that do not address its network-level organization [61].

### 6.2. Multi-Target Therapeutic Strategies

The recognition of LOAD as a network disorder provides a strong rationale for multi-target therapeutic strategies, involving either rational drug combinations or single agents designed to modulate more than one pathological process. Therapies that simultaneously influence Aβ, tau, neuroinflammation, synaptic dysfunction, or proteostatic failure are, in principle, better aligned with the biology of disease progression than monotherapies directed at a single target [15,19,142]. Representative investigational agents directed at neuroinflammation, tau hyperphosphorylation, synaptic dysfunction, and metabolic and bioenergetic alterations are illustrated in Figure 4.

Preclinical studies indicate that combined targeting of Aβ and phosphorylated tau can reduce multiple pathological features more effectively than isolated interventions. Therapeutic approaches aimed at modulating microglial phenotypes, restoring lipid homeostasis, or enhancing lysosomal and autophagic function may similarly complement amyloid- or tau-directed strategies by acting on shared regulatory nodes; future disease modification will likely require pathway combinations tailored to the dominant biology of each disease stage [191].

This framework also helps explain why some apparently “downstream” targets may still be clinically relevant. Interventions that preserve synaptic integrity, improve glial metabolism, or restore proteostasis may not eliminate the initiating pathology but could still reduce network fragility and slow functional decline—shifting the therapeutic goal from suppressing a single lesion to rebalancing an interconnected biological system [192].

### 6.3. Precision Medicine in LOAD

Precision medicine in LOAD will likely depend on integrating genetics, fluid biomarkers, imaging, and clinical phenotyping into pathway-informed patient stratification models. Recent advances in polygenic risk scoring support the idea that aggregated genetic burden improves risk estimation beyond APOE alone, although prediction remains affected by ancestry, cohort composition, and the biological interpretation of non-coding loci [193]. Cell-type-specific polygenic approaches have begun to link genetic risk more directly to microglial, astrocytic, and other cellular processes, suggesting that future stratification models may capture biologically distinct disease subtypes rather than only global risk [194]. Biomarker development is expanding in parallel: amyloid PET, tau PET, CSF biomarkers, plasma phospho-tau, and proteomic signatures are increasingly capable of identifying specific pathological axes and their temporal sequence, and integration of amyloid and tau imaging with proteomics and genomics has already begun to reveal biologically meaningful subgroups that differ in rate of progression, pathway dominance, and therapeutic response [195,196].

Taken together, these developments suggest that precision medicine in LOAD should not be framed as a purely genetic enterprise, but rather as a multimodal, pathway-oriented strategy in which genetic background, molecular biomarkers, and clinical phenotype are jointly used to define disease mechanisms at the individual level [197].

## 7. Future Directions and Research Perspectives

Future progress in LOAD research will depend on moving beyond pathway description toward mechanistic integration. Although GWASs and sequencing studies have identified an expanding repertoire of risk loci, a major challenge remains the functional interpretation of how these variants influence cellular behavior across disease stages; many non-coding loci remain poorly understood, and their effects are likely to be highly context-dependent, varying across cell type, age, and disease state [26].

One major priority is the integration of genetics with single-cell and spatial multi-omics. Recent human brain atlases and cell-type-specific polygenic studies have shown that genetic risk is concentrated in specific cellular compartments—particularly microglia, astrocytes, and other components of the neurovascular and glial environment—and are beginning to identify regulatory programs connecting inherited risk to transcriptional, epigenetic, and proteomic changes in disease-relevant cells [194].

A second major direction involves multimodal biomarker integration. The combination of PET imaging, CSF and plasma proteomics, genomics, and longitudinal clinical phenotyping offers an increasingly powerful framework for defining disease trajectories and identifying biologically coherent subtypes; large-scale resources, including harmonized proteomic consortia and integrated omics-imaging studies, may accelerate the discovery of biomarkers that reflect pathway activity rather than only global disease status [196].

Future therapeutic development should also increasingly adopt systems biology principles, incorporating molecular stratification, pathway-specific enrichment, and combination strategies designed around interacting mechanisms rather than testing isolated targets in biologically heterogeneous populations—a transition that will be especially important for translating modest biomarker success into durable clinical benefit [186].

Finally, a critical unresolved issue is generalizability, discussed further in Section 8: many genetic and biomarker models have been developed primarily in populations of European ancestry or in highly selected trial cohorts, raising concerns about transferability across ancestries, comorbidity patterns, and real-world clinical settings [198].

In summary, the future of LOAD research lies in linking genetic architecture to cell-type-specific biology, multimodal biomarkers, and mechanism-based therapeutic design—an approach likely essential for moving from descriptive models of disease complexity toward clinically actionable frameworks.

## 8. Limitations of the Review

As a narrative review, this article does not follow a formal systematic review protocol, and no quantitative synthesis or meta-analysis was performed. The selection and interpretation of the included literature may therefore be influenced by author judgment and may not capture all available evidence. To mitigate the resulting risk of selection bias, the genes and variants discussed in detail were selected using explicit, pre-specified criteria (Section 2), drawing primarily on the largest available GWAS meta-analyses rather than on individual candidate-gene studies. The reviewed studies also vary substantially in population ancestry, study design, genotyping methods, biomarker assessment, and clinical outcomes, which limits direct comparability across findings; much of the available genetic evidence in particular is derived from populations of European ancestry, reducing the generalizability of pathway-based interpretations to more diverse populations. Finally, although this review integrates genetic and mechanistic evidence into a systems-level framework, many proposed pathway interactions—including the AMPK–tau relationship and the boundary between amyloid-dependent and amyloid-independent (PART-like) tau pathology discussed in Section 5.1—remain incompletely validated in longitudinal human studies.

## 9. Conclusions

Late-onset Alzheimer’s disease is increasingly recognized as a complex, multifactorial disorder driven by the convergence of genetic susceptibility and interconnected molecular pathways. Rather than reflecting a single pathogenic cascade, current evidence supports a network-based model in which amyloid processing, tau pathology, lipid metabolism, immune activation, proteostasis, and mitochondrial function interact dynamically to shape disease onset and progression. Within this framework, key pathogenic axes—including the amyloid–tau, lipid–immune, inflammation–proteostasis, and mitochondria–synaptic dysfunction axes—illustrate how genetic risk factors such as *APOE*, *TREM2*, and *ABCA7* do not act in isolation, but converge on a limited set of shared biological pathways to drive neurodegeneration.

From a translational perspective, this integrated view offers a mechanistic explanation for the limited efficacy of single-target therapies and supports a shift toward multi-target and pathway-oriented therapeutic strategies. The integration of genetic profiling with multimodal biomarkers and pathway-based patient stratification may accelerate the development of precision medicine approaches, enabling more individualized and biologically informed interventions. Realizing this potential will depend on continued progress in single-cell and spatial multi-omics, larger and more ancestrally diverse genetic and biomarker cohorts, and longitudinal studies capable of validating the pathway interactions proposed in this review.

In conclusion, framing LOAD as a systems-level network disorder—rather than a linear sequence of isolated pathogenic events—provides a more coherent and clinically actionable framework for future research. Targeting the convergence of molecular pathways, rather than individual pathological features in isolation, represents a critical step toward durable disease-modifying strategies in LOAD.

## Data Availability

No new data were created or analyzed in this study. Data sharing is not applicable to this article.
